# Supplementary material for: Predicting visual function by interpreting a neuronal wiring diagram
Source: Nature. 2024 Oct 2;634(8032):113–23. doi: 10.1038/s41586-024-07953-5 (PMC11446822; doi:10.1038/s41586-024-07953-5)
Supplement: Supplementary file 6 — CRF and ERF predictions for individual TmY4 and TmY9 cells. Analogous to Supplementary Data 3, but for TmY target types. Shown are the top four monosynaptic pathways, the strongest pathway passing through each of the top ten intermediary types (ranking from Extended Data Fig. 7), and the trisynaptic pathway Tm1–TmY–Dm3–TmY (see the section entitled Prediction of spatial normalization). [file 41586_2024_7953_MOESM6_ESM.zip › DataS4/TmY9q/720575940618163520.pdf]

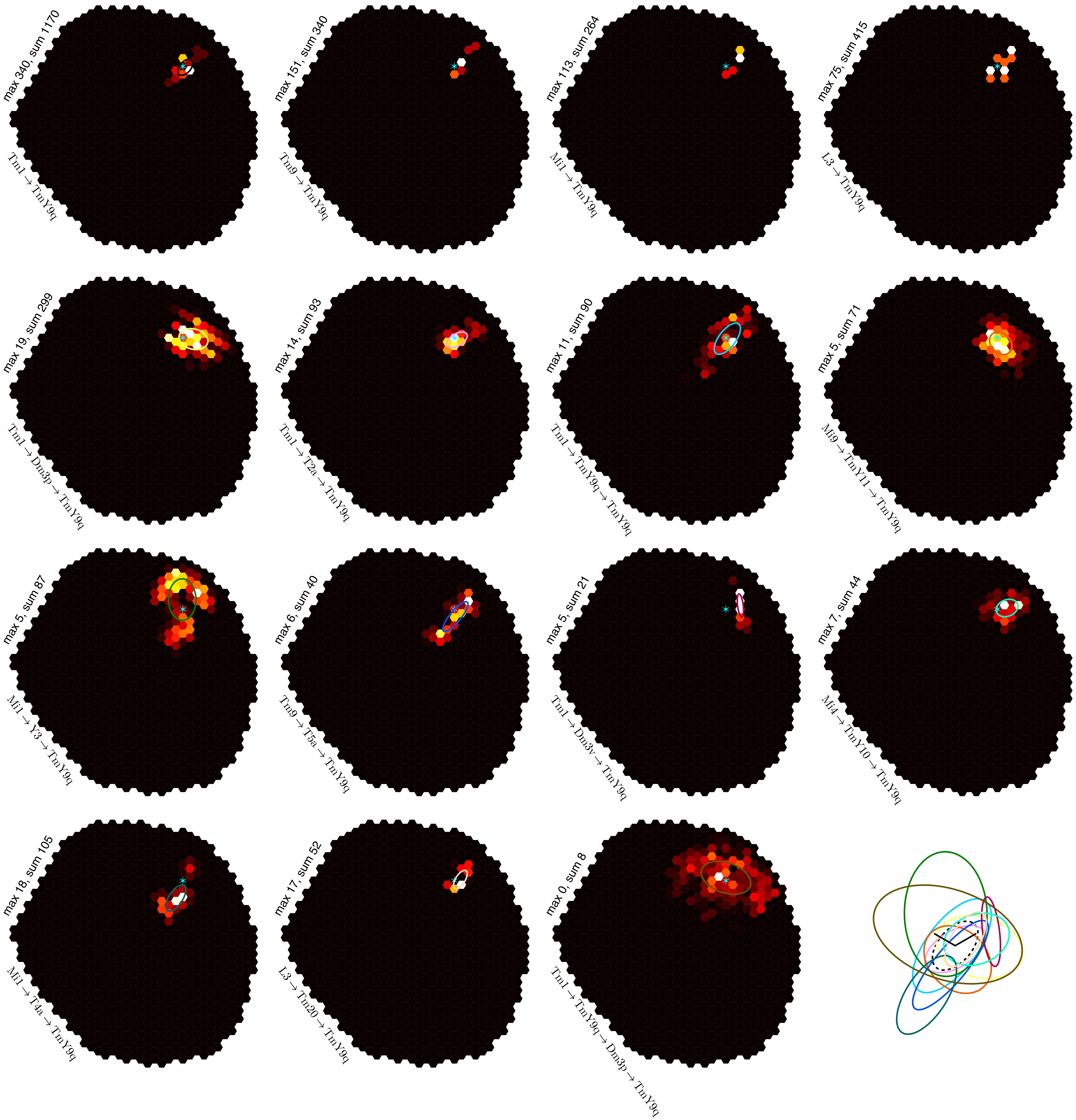
max 340, sum 1170
Tm1 → TmY9q
max 151, sum 340
Tm9 → TmY9q
max 113, sum 264
Mi1 → TmY9q
max 75, sum 415
L3 → TmY9q
max 19, sum 299
Tm1 → Dm3p → TmY9q
max 14, sum 93
Tm1 → T2a → TmY9q
max 11, sum 90
Tm1 → TmY9q → TmY9q
max 5, sum 71
Mi9 → TmY11 → TmY9q
max 5, sum 87
Mi1 → Y3 → TmY9q
max 6, sum 40
Tm9 → T5a → TmY9q
max 5, sum 21
Tm1 → Dm3v → TmY9q
max 7, sum 44
Mi4 → TmY10 → TmY9q
max 18, sum 105
Mi1 → T4a → TmY9q
max 17, sum 52
L3 → Tm20 → TmY9q
max 0, sum 8
Tm1 → TmY9q → Dm3p → TmY9q
